# Interaction of *Hm*C1q with leech microglial cells: involvement of C1qBP-related molecule in the induction of cell chemotaxis

**DOI:** 10.1186/1742-2094-9-37

**Published:** 2012-02-22

**Authors:** Muriel Tahtouh, Annelise Garçon-Bocquet, Françoise Croq, Jacopo Vizioli, Pierre-Eric Sautière, Christelle Van Camp, Michel Salzet, Patricia Nagnan-le Meillour, Joël Pestel, Christophe Lefebvre

**Affiliations:** 1 Laboratoire de Spectrométrie de Masse Biologique Fondamentale et Appliquée - EA4550, Microglial activation group, Université Lille Nord de France, Université Lille 1, IFR 147, bâtiment SN3, 59655, Villeneuve d'Ascq, France; 2 INRA, CNRS-UMR 8576, Unité de Glycobiologie Structurale et Fonctionnelle, IFR 147, 59655 Villeneuve d'Ascq, France; 3 CNRS-UMR 8576, Unité de Glycobiologie Structurale et Fonctionnelle, IFR 147, Université Lille Nord de France, Université Lille 1, 59655, Villeneuve d'Ascq, France

**Keywords:** C1q, C1qBP (alias gC1qR), Chemotaxis, Medicinal leech, Microglia, Nerve repair, Neuroinflammation

## Abstract

**Background:**

In invertebrates, the medicinal leech is considered to be an interesting and appropriate model to study neuroimmune mechanisms. Indeed, this non-vertebrate animal can restore normal function of its central nervous system (CNS) after injury. Microglia accumulation at the damage site has been shown to be required for axon sprouting and for efficient regeneration. We characterized *Hm*C1q as a novel chemotactic factor for leech microglial cell recruitment. In mammals, a C1q-binding protein (C1qBP alias gC1qR), which interacts with the globular head of C1q, has been reported to participate in C1q-mediated chemotaxis of blood immune cells. In this study, we evaluated the chemotactic activities of a recombinant form of *Hm*C1q and its interaction with a newly characterized leech C1qBP that acts as its potential ligand.

**Methods:**

Recombinant *Hm*C1q (r*Hm*C1q) was produced in the yeast *Pichia pastoris*. Chemotaxis assays were performed to investigate r*Hm*C1q-dependent microglia migration. The involvement of a C1qBP-related molecule in this chemotaxis mechanism was assessed by flow cytometry and with affinity purification experiments. The cellular localization of C1qBP mRNA and protein in leech was investigated using immunohistochemistry and *in situ *hybridization techniques.

**Results:**

r*Hm*C1q-stimulated microglia migrate in a dose-dependent manner. This r*Hm*C1q-induced chemotaxis was reduced when cells were preincubated with either anti-*Hm*C1q or anti-human C1qBP antibodies. A C1qBP-related molecule was characterized in leech microglia.

**Conclusions:**

A previous study showed that recruitment of microglia is observed after *Hm*C1q release at the cut end of axons. Here, we demonstrate that r*Hm*C1q-dependent chemotaxis might be driven via a *Hm*C1q-binding protein located on the microglial cell surface. Taken together, these results highlight the importance of the interaction between C1q and C1qBP in microglial activation leading to nerve repair in the medicinal leech.

## Background

In the mammalian central nervous system (CNS), microglial cells constitute the resident immune cells, maintaining the integrity of the nervous system and able to respond to any kind of brain damage [1]. In healthy brain, resting microglial cells show a ramified morphology [2]. Under pathophysiological conditions, they rapidly change their morphology and change to amoeboid activated microglia. This activation is controlled by 'on' or 'off' signals [3]. Complement proteins are potential candidates to exert such 'on' signals on microglia and can induce neuronal cell death [4]. Indeed, the complement system can be activated by three different pathways, the classical, the lectin-dependent and the alternative pathways [4]. Moreover, following human brain infection or injury, production of complement by resident cells has been clearly demonstrated to be highly increased upon activation [5]. Interestingly, C1q, the first component of the classical complement pathway, may serve as a reliable marker of microglial activation, ranging from undetectable levels of C1q biosynthesis in resident microglia to high C1q expression in activated, non-ramified microglia. C1q synthesized and released by activated microglia has been shown to maintain and regulate microglial activation in diseased CNS tissue [4,6,7]. Thus, C1q plays an important role in microglia regulation after nerve injury.

Unlike mammals, the medicinal leech *Hirudo medicinalis *can fully regenerate its CNS after injury and restore function of individual neurons [8,9]. For this reason, the leech CNS, which combines simplicity and well known organization [10], has been an attractive model in neurobiology for decades. After injury, leech microglia immediately move toward the lesion site. This phenomenon has been shown to be essential to promote axon sprouting and successful nervous system repair [11-14]. Leech microglial cells exhibit morphological changes similar to vertebrate ones in the course of migration in response to tissue damage [15,16]. In our laboratory, we were interested to assess the role of C1q in microglial cell accumulation after leech CNS injury.

We previously characterized, for the first time in an invertebrate nervous system, a C1q domain-containing (C1qDC) factor named *Hm*C1q [17]. Of interest, its involvement in leech microglia recruitment following experimental injury has been clearly demonstrated. In order to study its interaction with CNS cells and elucidate its role in microglial cell chemotaxis, the recombinant form of *Hm*C1q (r*Hm*C1q) was produced in the yeast *Pichia pastoris*. In the present report, we demonstrate the chemotactic activity of the recombinant protein on leech microglial cells and we used r*Hm*C1q to tightly explore its functions in the leech nervous system following trauma. In vertebrates, C1q has been demonstrated to exert its chemotactic activity through C1q receptors expressed on immune cells [18]. Finally, the interaction between r*Hm*C1q and leech CNS cells was investigated, allowing the identification of a C1qBP-related molecule, which was named *Hm*C1qBP, homologous to the mammalian C1q receptor (alias gC1qR, p32, p33, C1qBP, HABP1; SF2p32, TAP) [19]. Therefore the involvement of a C1q domain-containing factor in microglial activation is demonstrated for the first time in a CNS.

## Methods

### Recombinant HmC1q production and purification

#### Expression vector construction

The cDNA encoding the *Hm*C1q (Genbank accession number EU581715) [17] was amplified by PCR from total leech CNS cDNA as template. Amplification was performed using specific forward (5'gcgccctacgtaatgaaagtatttctggaaatcctcgc3') and reverse (5'taattgcggccgctcactttctgcttgcaatt3') primers containing *Sna*BI (forward, bold) and *Not*I (reverse, bold) restriction sites, respectively, together with the predicted natural signal peptide sequence (forward, underlined). PCR amplifications were carried out on a Thermal Cycler (Eppendorf, Hamburg, Germany) with 150 ng of cDNA in a solution containing 1.25 U of Hot Start Proofreading DNA polymerase (Accu Prime™ Pfx, Life Technologies, Grand Island, NY, USA), 0.3 μM of each PCR primer, 1 × DNA polymerase manufacturer's buffer containing deoxyribonucleotide triphosphate (dNTP) in a final volume of 50 μl. The reaction cycles were performed as follows: 95°C for 2 minutes, followed by 35 cycles of 15 s at 95°C, 30 s at 60°C and 1 minute at 68°C. A single PCR product was obtained and ligated into the *Sna*BI and *Not*I digested pPIC3.5 K vector with T4 DNA Ligase (Life Technologies, Grand Island, NY, USA) according to the instructions of the Multi-Copy *P. pastoris *Expression Kit manual (Life Technologies, Grand Island, NY, USA). The plasmid DNA *Hm*C1q/pPIC3.5 K was amplified into *Escherichia coli *Top10F' chemically competent cells (Life Technologies, Grand Island, NY, USA). Cloning steps were verified by both strands DNA sequencing (Eurogentec S.A., Liege, Belgium).

#### Transformation of P. pastoris strain and screening for protein expression

The recombinant plasmid *Hm*C1q/pPIC3.5 K (see above) was linearized with *Sac*I and used to transform GS115 *P. pastoris *strain by electroporation according to the method described in the manufacturer's manual (Life Technologies, Grand Island, NY, USA). Selection of His+/Mut+ transformants was achieved as previously described [20]. The recombinant clones were screened on yeast/peptone/dextrose (YPD) agar plates containing growing doses of G418 (Geneticin; Life Technologies, Grand Island, NY, USA) for the presence of multiple inserts. A total of 20 clones were inoculated in 10 ml of buffered glycerol-complex (BMGY) medium (1% w/v yeast extract, 2% w/v peptone, 1.34% w/v yeast nitrogen base, 4 μg/ml D-biotin, 100 mM potassium phosphate, pH 6.0, 1% v/v glycerol) and incubated at 29°C and 225 rpm. After 48 h, cells were pelleted by centrifugation for 2 minutes at 1,000 *g *at room temperature (RT). The pellets were gently resuspended in 2 ml of basal minimum medium (BMM) (1.34% w/v yeast nitrogen base, 4 μg/ml D-biotin, 100 mM potassium phosphate, pH 6.0, 0.5% v/v MeOH) and incubated at 29°C and 225 rpm. After 48 h, the cultures were centrifuged (10,000 *g*, 10 minutes, 4°C), and the supernatants were dried under vacuum to be checked for protein expression by western blot. The clone corresponding to the highest production of protein was stored in glycerol at -80°C.

### Purification of recombinant protein

Aliquots of supernatants obtained from 32 cultures of 2 ml were centrifuged at 12,000 *g *for 10 minutes at 4°C, filtered through a 0.8 μm filter and concentrated until a 1 ml volume (Centricon YM-10, Millipore, Billerica, MA, USA). Purification was achieved in one step by reverse-phase high performance liquid chromatography (RP-HPLC) with a C_8 _column (250 × 4.1 mm, Grace-Vydac, Columbia, MD, USA) with a linear gradient of acetonitrile (ACN) in acidified water (0,1% trifluoroacetic acid) from 2% to 32% ACN for 60 minutes at a flow rate of 1 ml/min. The presence of r*Hm*C1q in the eluted fractions was checked by western blotting. The RP-HPLC fraction containing the recombinant protein was dried under vacuum and stored at -20°C.

For further analyses, the transformed *P. pastoris *culture supernatant, the RP-HPLC-purified recombinant protein and the non-transformed *P. pastoris *culture supernatant will be respectively referred to as 'r*Hm*C1q supernatant', 'purified r*Hm*C1q' and 'control supernatant'.

### Western blotting

Samples (either r*Hm*C1q supernatant, control supernatant or purified r*Hm*C1q) were reconstituted in Laemmli buffer before loading onto a 12% acrylamide running gel and a 4% acrylamide stacking gel as previously described [21]. Briefly, migration was carried out using a cathode buffer (0.6% Tris base, 2.5% taurine, and 0.1% SDS) and an anode buffer (0.6% Tris base, 2.8% glycine, and 0.1% SDS). Gels ran at 70 V for 15 minutes and at 120 V for 45 minutes. Separated proteins were transferred to Nitrocellulose Transfer Membrane Protran BA 83 (Schleicher & Schuell Bioscience, Dassel, Germany) by electroblotting. After preincubation in blocking solution (BS) (phosphate-buffered saline (PBS) containing 0.05% Tween 20 and 2% ovalbumin fraction V) membranes were incubated overnight at 4°C with either rabbit polyclonal anti-*Hm*C1q antibody or preimmune serum (dilution 1:1,000 in BS). Specific rabbit polyclonal anti-*Hm*C1q antibodies were raised using a synthetic peptide corresponding to predicted His^197^-Thr^212 ^region of *Hm*C1q protein (Agro-bio, La Ferté Saint Aubin, France) [17]. After three PBS washes, goat anti-rabbit or anti-mouse IgG antibodies conjugated with horseradish peroxidase (dilution 1:20 000 in BS) (Jackson Immunoresearch, West Grove, PA, USA) were added for 1 h at RT. The final washes were performed in PBS and immunolabelled proteins were revealed with the ECL Kit SuperSignal West Pico Chemoluminescent Substrate (Thermo Fisher Scientific, Rockford, IL, USA) and Kodak X-Omat LS film (Sigma-Aldrich, St. Louis, MO, USA).

### Leech CNS and microglial cell preparation

All protocols regarding the use of leeches were carried out in strict accordance with the French legislation and European Treaty, and were in compliance with the Helsinki Declaration. *H. medicinalis *adult leeches were obtained from Ricarimpex (Eysines, France). The leech nerve cord (CNS) is constituted of 23 metameric ganglia joined by structures, called connectives, containing the axonal processes and glial cells [10]. After anesthesia in 10% ethanol at 4°C for 15 minutes, animal CNSs were dissected out in a sterile Ringer solution (115 mM NaCl, 1.8 mM CaCl_2_, 4 mM KCl, 10 mM Tris maleate, pH 7.4) under a laminar flow hood After isolation, samples were placed in three successive baths of antibiotics (100 UI/ml penicillin, 100 μg/ml streptomycin and 100 μg/ml gentamycin) for 15 minutes and further incubated in Leibovitz L-15 medium (Life Technologies, Grand Island, NY, USA) containing 2 mM L-glutamine, 0.6% glucose and 10 mM 4-(2-hydroxyethyl)-1-piperazineethanesulfonic acid (HEPES) (complete medium). The experimental injury was performed by crushing the connectives between the third and fourth ganglia. Nerve cords were used for *ex vivo *recruitment assays, whole mount immunohistochemistry, fluorescence *in situ *hybridization or nerve cell preparation.

For total nerve or microglial cell isolations, nerve cords treated as indicated above were placed in 35 mm Petri dishes with 200 μl of complete L-15 medium. Each ganglion was carefully decapsulated by removing the collagen layer enveloping the nerve cord with microscissors. Nerve cells, neurons and microglial cells were mechanically resuspended by gentle scraping (total nerve cells). After a filtration through 7 μm nylon mesh as described [17,22], the enriched microglial cell population was then collected and centrifuged at 1,000 *g *for 10 minutes at RT. The cell pellet was resuspended in L-15 medium (100 μl per nerve cord) for migration assays.

### Chemotaxis assays

*In vitro *chemotaxis assays were performed by using the double-P assay as described by Köhidai and colleagues with minor modifications [23]. Petri dishes (35 mm) were filled with 1 ml of a 0.5% agar and 1% gelatin solution. After drying, two 6 mm diameter wells were made, each one presenting a parallel individual channel. One well was filled with 50 μl of purified microglial cells (see above) and the other one with chemotactic factors or negative controls reagents. A channel was further created perpendicularly to others using a coverslip. By 1 h later, cells in the chemoattractant containing well were collected. Either r*Hm*C1q supernatant (0.1, 1, 3, 8 and 15 μl) or control supernatant (0.1, 1, 3, 8 and 15 μl) were used as chemotactic factors. For inhibitory chemotactic experiments, cells were preincubated for 1 h at RT either with rabbit polyclonal anti-*Hm*C1q antibody or with preimmune serum as negative control (both 1:250); and either with rabbit polyclonal anti-human C1qBP antibody (1:1,000) or with rabbit IgG isotype as negative control (1:1,000). The number of migrating cells was counted on a hemocytometer (three different counts) under Axioskop microscope (Zeiss, Oberkochen, Germany). Additional experiments were also performed with RP-HPLC-purified r*Hm*C1q as chemoattractant in similar conditions. Experiments were performed in triplicate. The results were expressed as the mean cell number ± SD. Comparisons between means were made using the Student's t test. Statistical differences were considered to be significant if *p *was < 0.01.

### *Ex vivo *microglial cell recruitment assays

Ganglia 2, 3, 4 and 5 were dissected from the animal and pinned in separate plastic 35 mm Petri dishes (Falcon 3005, Becton Dickinson, Franklin Lakes, NJ, USA) coated with silicone rubber (Sylgard 184, Dow Corning Corp., Midland, MI, USA) and placed in L-15 complete medium. The following products were respectively injected (8 μl) inside the connectives separating the ganglia 3 and 4: PBS; r*Hm*C1q supernatant; r*Hm*C1q supernatant + anti-*Hm*C1q antibody (dilution 1:5,000); r*Hm*C1q supernatant + preimmune serum (dilution 1:5,000) or the control yeast supernatant. For injections, patch pipettes were pulled from borosilicate glass capillaries (outer diameter 1.5 mm, Clark GC 150 F-10) using a two-stage horizontal micropipette puller (model P-97, Sutter Instrument Co., Novato, CA, USA) (pipette resistance 3 to 5 MΩ). The connectives were crushed immediately after injection with fine forceps on both side of the injection site and the tissues were fixed in buffered 4% paraformaldehyde, pH 7.4 4 h after the injection. The Hoechst 33342 (Life Technologies, Grand Island, NY, USA) fluorescent dye (dilution 1:1,000 in L-15 medium) was then applied to injured nerve cords for 30 minutes to counterstain the nuclei of microglial cells. Microglial cells movement in response to these different injections was then observed with an inverted microscope (DMIRE2, Leica Microsystems, Wetzlar, Germany).

### Immunohistochemistry

In experiments with anti-human C1qBP antibody, analyses were performed on nerve cords dissected out as described above and incubated 6 h in complete L-15 medium. They were fixed for 1 h at 4°C, immediately after dissection (T0) or 6 h (T6h) and 24 h (T24h) after incubation in complete L-15 medium, in 4% paraformaldehyde, washed in PBS, permeabilized by a 24 h-incubation at RT in 1% Triton X100 in PBS and preincubated for 8 h at RT in 1% Triton, 3% normal donkey serum (NDS) and 1% ovalbumin in PBS. Samples were then incubated overnight at 4°C with specific rabbit polyclonal anti-human C1qBP antibody (1:250) diluted in a PBS solution containing 1% bovine serum albumin (BSA), 0.05% Triton, 1% NDS and 1% ovalbumin (AB solution). After three washes with PBS, samples were incubated 1 h at room temperature with anti-rabbit donkey antibody (Life Technologies, Grand Island, NY, USA) conjugated to Alexa Fluor 488 (1:2,000 in the AB solution), rinsed with PBS and finally mounted with Glycergel (Sigma-Aldrich, St. Louis, MO, USA). Prior to mounting, the cell nuclei were counterstained by Hoechst dye as previously described. Samples without the addition of primary antibody were used as negative control. Slides were kept at 4°C in the dark until observation, realized with a Zeiss LSM780 confocal microscope (Zeiss, Oberkochen, Germany). We opted to present microglia nuclei in white for a better display of the results.

### Fluorescent *in situ *hybridization (FISH)

Nerve cords were fixed for 1 h at 4°C in 4% paraformaldehyde just after dissection. The 5' biotin-labeled specific antisense probe and sense probe (negative control) were generated from a specific sequence (corresponding to the nucleotide sequence 154 to 859 of C1qBP molecule; Genbank accession number JN207836). After PCR amplification and the insertion of the product in pGEM-T easy vector system (Promega, Madison, WI, USA), the RNA sequence of interest was obtained by *in vitro *transcription using DIG/Biotin RNA-labeling kit according to the manufacturer's instructions (Roche Diagnostics, Rotkreuz, Switzerland). The hybridization protocol was performed as previously described [24]. Nerve cords were incubated with a secondary anti-biotin antibody conjugated to Alexa Fluor 488 (dilution 1:5,000 in PBS) (Life Technologies, Grand Island, NY, USA). Final rinsing and mounting steps for confocal microscopy observation were performed as described above.

### Flow cytometry analyses

Total nerve cells isolated as described above from eight nerve cords were equally distributed (about 10^6 ^cells per tube) and kept for 24 h at RT in L-15 medium either alone or with purified r*Hm*C1q. Then, they were incubated for 30 minutes with the fluorescein-labeled mouse monoclonal anti-human C1qBP (60.11) antibody (Santa Cruz Biotechnology, Santa Cruz, CA, USA) (dilution 1:250 in L-15 medium). Nerve cells were then washed with L-15 medium, centrifuged for 8 minutes at 1,000 *g *at 4°C. Cell pellet was resuspended in 500 μl L-15 medium and finally, examined by fluorescence-activated cell sorter (FACS) (EPICS XL4-MCL, Beckman Coulter, Diagnostics division, Brea, CA, USA) equipped with an argon ion laser with an excitation power of 15 mW at 488 nm. Forward scatter (FSC) and side scatter (SSC) were analyzed on linear scales, while green (FL1) was analyzed on logarithmic scales. Data acquisition and analysis were performed using Expo32 software.

### Microglial cell protein extraction

Protein extraction was performed by trichloroacetic acid/acetone precipitation and was resuspended in lysis buffer (7 M urea, 2 M thiourea, 4% 3-[(3-cholamidopropyl)dimethylammonio]-1-propanesulfonate (CHAPS)). Protein extracts were desalted by Zeba Desalt Spin Columns, 2 ml (Pierce) following the manufacturer's guidelines. Protein concentration was determined using the Bradford method (Bio-Rad, Hercules, CA, USA) and protein extracts were stored at -20°C.

### Human C1q biotinylation and streptavidin affinity purification

The biotinylation of the recombinant human C1q (Prospecbio, Rehovot, Israel) was carried out by using the sulfo-NHS-SS-biotin kit (Thermo Fisher Scientific, Rockford, IL, USA) according to the manufacturer's instructions. Briefly, proteins were biotinylated in PBS with a 20-fold molar excess for 30 minutes at RT. Unreacted sulfo-NHS-SS-biotin was removed using the Zeba Desalt Spin Columns (Thermo Fisher Scientific, Rockford, IL, USA). Biotinylated human C1q was immediately fixed onto a streptavidin column (Thermo Fisher Scientific, Rockford, IL, USA), previously equilibrated with five volumes of PBS 0.1 M. The interaction between biotin and streptavidin occurred at RT for 10 minutes. Microglia protein extract (800 μg) was added in the column, incubated overnight at 4°C and rinsed ten times with PBS 0.1 M. Captured microglial cell proteins were eluted from the streptavidin-agarose with 5% 2-mercaptoethanol/PBS 0.1 M at 30°C for 30 minutes. Proteins were precipitated in 10% trichloroacetic acid/acetone at -20°C for 45 minutes, and centrifuged at 13,000 *g *for 15 minutes. The protein pellet was washed in cold acetone, air dried and dissolved in Laemmli buffer. Two other columns were used for the negative controls: the first one containing the biotinylated human C1q with no microglia protein extract and the second one containing only the microglia protein extract to evaluate the unspecific reaction between streptavidin and microglial cell components. Samples were loaded on a 12% SDS-PAGE gel and analyzed by western blotting using a rabbit polyclonal anti-human C1qBP antibody (Santa Cruz Biotechnology, Santa Cruz, CA, USA), diluted at 1:5,000 in BS (see above).

## Results

### Production of recombinant HmC1q in *P. pastoris*

**Figure 1:**
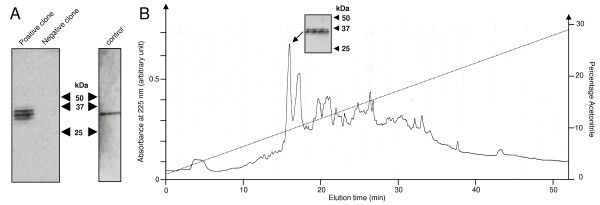
**Expression of r*Hm*C1q in the *Pichia pastoris *yeast host system**. **(A) **Immunodetection of r*Hm*C1q (selected positive clone) by western blot with the polyclonal anti-*Hm*C1q antibodies is compared with the native *Hm*C1q released in leech central nervous system (CNS)-conditioned medium (control). Supernatant from non-transformed yeast culture was used as a negative control. **(B) **Part of the chromatogram of the yeast culture illustrating the purification of r*Hm*C1q by reverse-phase high performance liquid chromatography (RP-HPLC). The r*Hm*C1q molecule was eluted at 10% of acetonitrile (see arrow) and was detected by western blot with the polyclonal anti-*Hm*C1q antibody.

*Hm*C1q was expressed by the yeast *P. pastoris *under control of the methanol-inducible alcohol oxidase promoter and secreted using its natural signal peptide. A positive clone was selected following the detection of a signal using the polyclonal anti-*Hm*C1q antibodies by western blot because of its relevance to the native *Hm*C1q detection as previously described (Figure 1A, control) [17]. No significant difference was detected between the recognition of r*Hm*C1q in the transformed yeast supernatant and native *Hm*C1q released in the conditioned medium (Figure 1A, positive clone vs control). The negative control performed with a non-transformed yeast clone did not show any immunoreactive bands. The presence of three bands on SDS-PAGE shows slight differences in the recombinant protein, suggesting different levels of glycosylation. The shared immunoreactivity suggests that r*Hm*C1q has the same size and migration profile as the native *Hm*C1q. The expression of r*Hm*C1q was observed up to 4 days without any significant proteolytic degradation (data not shown). The protein was purified by RP-HPLC and eluted as the major peak at 10% of acetonitrile (arrow in Figure 1B). The corresponding molecule (purified r*Hm*C1q) was then analyzed by western blot by using the same antiserum and displayed specific immunoreactivity (Figure 1B).

### *In vitro *chemotactic activity of r*Hm*C1q on leech microglial cells

**Figure 2:**
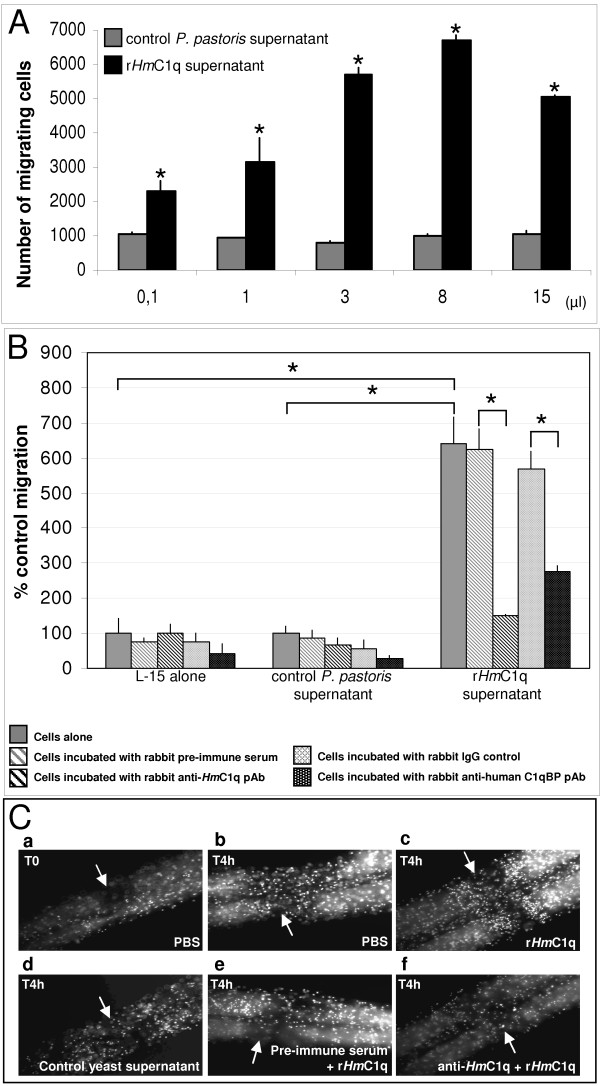
**Chemotactic effect of r*Hm*C1q on leech microglia migration**. **(A) **r*Hm*C1q supernatant was compared to control supernatant using similar volumes in respective chemotaxis assays (0.1, 1, 3, 8, 15 μl). The diagram illustrates the chemotactic activity of r*Hm*C1q in a dose-dependent manner. Asterisks denote cell migration of the indicated sample that is significantly different (*p <*0.01) than the corresponding control value. **(B) **Inhibitory effects on r*Hm*C1q-mediated leech microglia chemotaxis. Leech microglial migration was evaluated with either L15 medium, control *Pichia pastoris *supernatant or r*Hm*C1q-containing supernatant as chemoattractant. An inhibitory effect was demonstrated by preincubation of cells either with rabbit polyclonal anti-*Hm*C1q antibodies (black hatching in each condition) or with a rabbit polyclonal anti-human C1qBP (alias gC1qR) antibody (black dots in each condition). Negative controls consisted of cells preincubated with rabbit preimmune serum or rabbit IgG isotype respectively. Asterisks denote cell migration of the indicated sample that is significantly different (*p <*0.01) than the corresponding controls. **(C) **Chemotactic effect of r*Hm*C1q on leech microglia in *ex vivo *assays. Microglial cells, which are the only circulating resident cells present in the connectives, were counterstained with Hoechst dye. The Hoechst dying was observed immediately after crush injury (see arrows) with injection of phosphate-buffered saline (PBS) (a) or 4 h after injury with injection of PBS (b) illustrating microglial accumulation at the lesion site. Injection of r*Hm*C1q increased microglial cells recruitment (c) compared either to the presence of PBS only (b) or to control yeast supernatant (d). When tissues were injected with polyclonal anti-*Hm*C1q antibody (f), the chemotactic activity of r*Hm*C1q was not observed whereas the corresponding preimmune serum did not exert any neutralizing effect (e).

Native *Hm*C1q has been shown to recruit leech microglial cells in a dose-dependent manner [17]. In the present study, the biological activity of the r*Hm*C1q supernatant was compared to the control supernatant one using similar volumes in respective chemotaxis assays (0.1, 1, 3, 8, 15 μl). Freshly prepared leech microglial cells were demonstrated to migrate towards r*Hm*C1q supernatant also in a dose-dependent manner. The optimal effect was observed by using 8 μl of r*Hm*C1q supernatant while a higher amount (15 μl) caused a decreased migration of the microglia. This effect is a characteristic of cytokines having chemotactic activity and has been described for other chemoattractant factors of leech CNS conditioned medium [22]. Of interest, similar dose-dependent results were obtained by using purified r*Hm*C1q (data not shown). The control supernatant used as negative control did not exert any significant effect on microglial cell recruitment (Figure 2A).

In order to maintain a relevant comparison between r*Hm*C1q and a pertinent negative control, we used the r*Hm*C1q supernatant (8 μl) as positive control and the control supernatant (8 μl) as negative control. Chemotaxis assays were performed using the r*Hm*C1q supernatant and two negative control media (L-15 and control *P. pastoris *supernatant) in the presence of blocking antibodies directed against the leech protein (anti-*Hm*C1q) and the human C1q receptor molecule (anti-C1qBP), respectively (Figure 2B). Compared to r*Hm*C1q supernatant, negative controls did not exert any significant chemotactic effect (Figure 2B).

Interestingly, the r*Hm*C1q supernatant-mediated microglial recruitment was inhibited by using rabbit polyclonal anti-*Hm*C1q antibodies (Figure 2B, black hatched bars) whereas no significant inhibitory effect was detected with the preimmune serum (Figure 2B, gray hatched bars). In addition, no effect was observed by preincubating cells with polyclonal anti-*Hm*C1q antibodies in negative controls (L-15 medium or the control *P. pastoris *supernatant). Finally, the involvement of an *Hm*C1q binding protein as receptor in mediating microglia chemotaxis in response to r*Hm*C1q was investigated. Pretreatment of microglial cells with rabbit polyclonal anti-human C1qBP antibodies significantly abrogated the chemotactic response to r*Hm*C1q (Figure 2B, black dotted bars) whereas no significant inhibitory effect was detected using rabbit IgG isotype as negative control (Figure 2B, gray dotted bars). No neutralizing effect was observed in negative control assays (L-15 or control supernatant) (Figure 2B).

Taken together, these results clearly indicate that leech microglial cells specifically respond to r*Hm*C1q in a dose-dependent manner and strongly suggest that *Hm*C1q-mediated recruitment is exerted through a C1qBP-related molecule.

### *Ex vivo *chemotactic effect of r*Hm*C1q on resident microglial cells in injured nerve cords

The *in vitro *chemotaxis assays were corroborated by the *ex vivo *experiments performed on whole collected and injured nerve cords. The cell movement was analyzed by Hoechst dye counterstaining because microglia are the only circulating resident cells present in connectives (Figure 2C). The microglial cell migration at the site of injury was evaluated 4 h after injection of different molecules in nerve cords, immediately followed by experimental lesion. In the positive control experiment, the microglial cell accumulation at the lesion site strongly increased after nerve injury (Figure 2C, a vs b). Interestingly, the injection of r*Hm*C1q supernatant enhanced the microglia recruitment compared to those obtained in the presence of PBS only (Figure 2C, b vs c) or in the presence of the control supernatant (Figure 2C, c vs d). Of interest, while preincubation with rabbit preimmune serum did not have any significant neutralizing effect on r*Hm*C1q-mediated recruitment (Figure 2C, c vs e), the injection of rabbit polyclonal anti-*Hm*C1q antibodies reduced the r*Hm*C1q supernatant-mediated chemotactic activity (Figure 2C, c vs f). These results specifically confirmed the capacity of r*Hm*C1q to increase *ex vivo *the microglial cell recruitment following injury, compared to a normal cell accumulation in the presence of PBS.

### Characterization of a leech C1qBP related molecule

**Figure 3:**
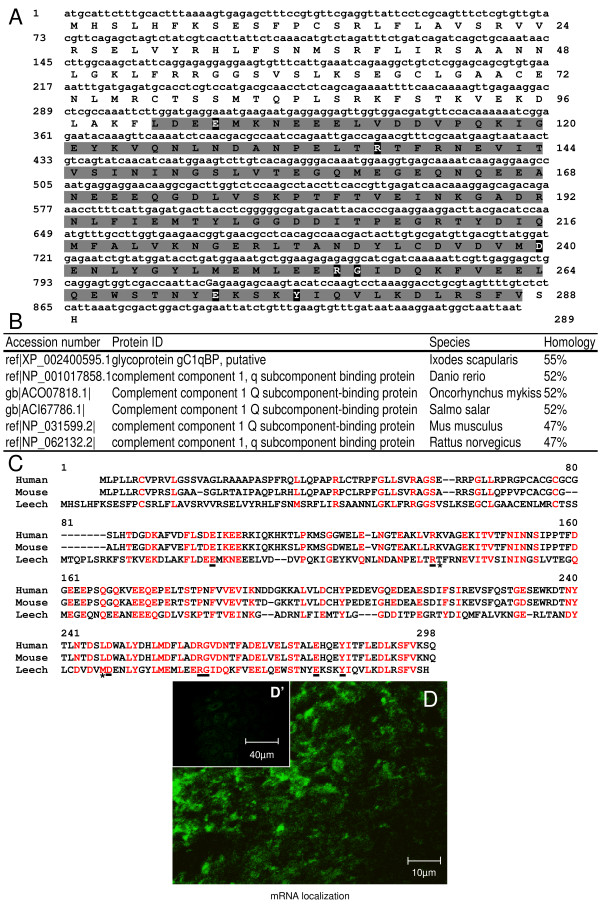
**Characterization of a C1qBP-related molecule (*Hm*C1qBP) in the medicinal leech**. **(A) **Nucleotide and amino acid sequences of leech *Hm*C1qBP containing the MAM33 conserved domain are highlighted in light gray (101 to 287). Numbers of nucleotides and amino acids are indicated on left and right of the sequence, respectively. Residues considered important for correct folding and receptor-ligand interaction in human and mouse C1qBP are highlighted in black. **(B) **Blast-P alignments show the most important homologies (ranging from 47% to 55%) between *Hm*C1qBP and some C1qBP molecules from other species. **(C) **Multiple alignments between human (NP_001203), mouse (NP_031599) and leech (Genbank accession number JN207836) C1qBP primary structures show numerous conserved residues (red). Black bars and asterisks indicate the nine structurally important residues cited above. **(D) **Fluorescence *in situ *hybridizations on freshly dissected nerve cord (T0) show localization of *Hm*C1qBP transcripts in microglial cells (arrows). **(D') **The insert corresponds to sense probe as a negative control.

Because of the neutralizing effect observed using anti-human C1qBP antibodies in chemotaxis assays (see above), the presence of a potential C1q-binding protein (receptor-like) was investigated in an expressed sequence tag (EST) library constructed from total RNA of nerve cords isolated from adult leeches. A full-length mRNA sequence for a C1q binding proteins (C1qBP) related molecule was identified (Genbank JN207836) encoding a 289-amino-acid sequence (Figure 3A). The theoretical molecular weight was predicted to be 32,973 Da according to the complete protein sequence. Similar to mammalian C1qBP, this sequence presents a MAM33 domain (Figure 3A) and contains the mature form as described for the human C1qBP [19]. Blast-P analyses of the leech sequence revealed homologies exclusively with many known C1qBP sequences (Figure 3B) with high conservation for the MAM33 domain [25,26].

Considering that only C1qBP molecules were matched from databases, whatever the rate of homology, we restricted the presentation of the Blast-P analysis to the highest homology percentages (ranging from 47% to 55%) in the table (Figure 3B). Multiple alignments were realized to show the similarities in primary structure of human, mouse and leech forms (Figure 3C). Interestingly, seven of the nine residues essential for conformation and ligand binding properties in the human C1qBP (Glu-89, Arg-122, Lys-123, Leu-231, Asp-232, Arg-246, Gly-247, Glu-264 and Tyr-268) are conserved in the leech sequence (Glu-104, Arg-136, Asp-240, Arg-254, Gly-255, Glu-272 and Tyr-276) [27]. These residues, which suggest comparable physicochemical features, are indicated in black boxes (Figure 3A) and are underlined in Figure 3C. The residues Lys-123 and Leu-231 in the human sequence are replaced by Thr-137 and Met-239 in the leech one, respectively (asterisks, Figure 3C). From all these elements, the leech molecule was named *Hm*C1qBP for *H. medicinalis *C1qBP.

### Localization of *Hm*C1qBP mRNA and protein in leech microglia

In order to specify the cell expressing the *Hm*C1qBP transcripts in the leech nervous system, specific fluorescence *in situ *hybridization (FISH) was carried out on injured nerve cords. The transcripts were mainly detected in the microglial cells of ganglia (Figure 3D) while no specific signals were detected with sense riboprobes used as negative control (Figure 3D').

**Figure 4:**
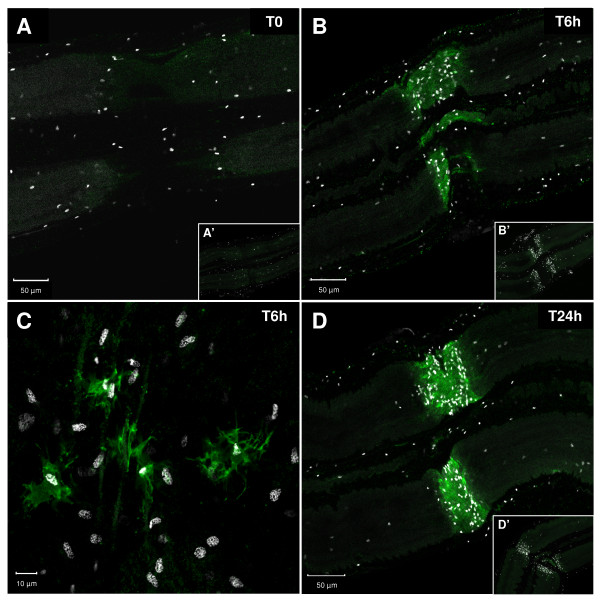
**Immunostaining of leech central nervous system (CNS) using rabbit polyclonal anti-human C1qBP antibodies (green)**. Ganglia were analyzed immediately **(A)**, 6 h **(B, C)**, or 24 h **(D) **following injury. Microglial cell nuclei (white) were stained with Hoechst fluorescent dye to observe cell migration. **(A) **No anti-C1qBP immunostaining was detected at the lesion site and no microglial accumulation was observed. **(B) **At 6 h following injury, positive immunostaining was observed at the site of crush. Simultaneously, microglial cells progressively accumulated at the lesion site. **(C) **High magnification image of the injury site. Since all microglia nuclei are shown by Hoechst counterstaining (white), the anti-C1qBP immunostaining exclusively detects only some microglial cells. **(D) **At 24 h following injury, the number of microglial cells is much greater at the lesion site and stronger positive immunostaining is observed. **(B', C', D') **No immunostaining was observed using secondary antibodies alone as negative controls.

Cells containing *Hm*C1qBP protein were investigated by immunohistochemistry with anti-human C1qBP antibodies on whole mounted injured nerve cords immediately after dissection, 6 h, or 24 h after lesion (Figure 4). The microglia recruitment was simultaneously studied by using a Hoechst dye (white nuclei). Immediately following the crush (T0), no immunostaining and no accumulation of microglial cells was observed (Figure 4A). At 6 h after injury C1qBP staining was detected at the lesion site of connectives and some microglial cells accumulated at this site (Figure 4B). Of interest, magnification of the injury site 6 h after injury showed that the C1qBP staining was exclusively located in some microglial cells among the whole recruited microglial population (Figure 4C). Therefore, the timecourse evolution of C1qBP staining is directly associated with the progressive migration of microglial cells. Interestingly, since some microglial cells are still C1qBP negative it suggests the existence of different states of reactivity in microglia (Figure 4C). The C1qBP signal is stronger 24 h after injury at the lesion site and is still correlated with the increase of accumulated microglia (Figure 4D). Negative controls performed using the secondary antibodies alone did not show any signal (Figure 4A',B',D'). This timecourse accumulation of C1qBP positive microglial cells highly suggests the involvement of C1qBP in the microglia recruitment.

### Identification of a C1qBP molecule on leech microglia

**Figure 5:**
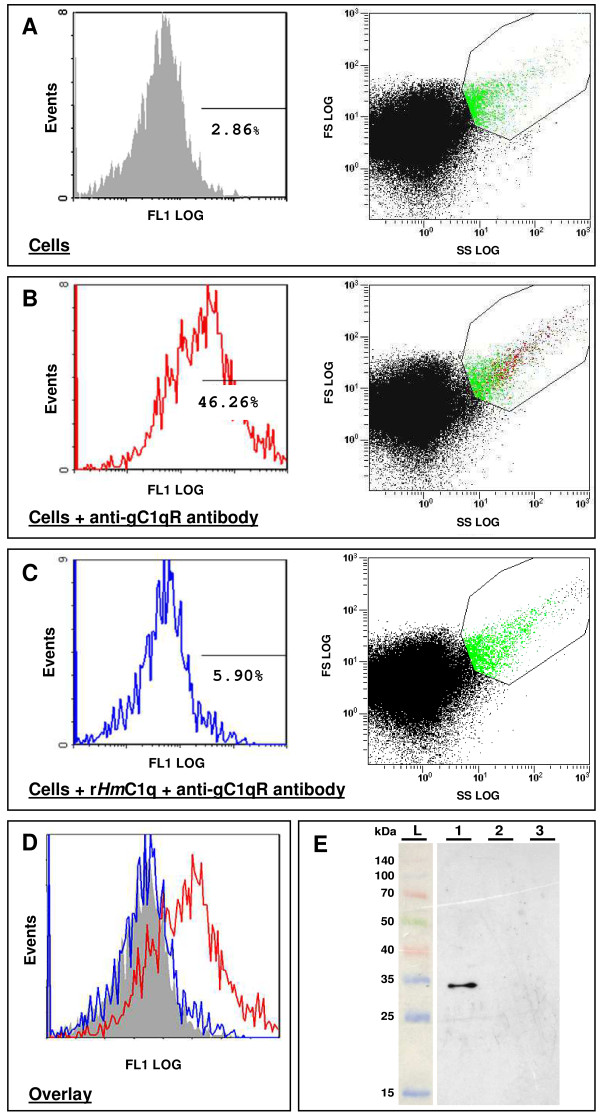
**Flow cytometry analysis of leech nerve cells using anti-human C1qBP antibody (A-D)**. **(A) **On the right side, we represent a forward scatter (FSC) versus side scatter (SSC) dot plot for leech nerve cells incubated overnight. Among the cells in the studied gate, 2.86% of autofluorescence cells are shown on the FL1 scale on the left. **(B) **On the right, we represent an FSC versus SSC dot plot for leech nerve cells incubated overnight alone, then treated with fluorescein-labeled mouse monoclonal anti-human C1qBP antibody for 30 minutes (dilution 1:250 in L-15 medium). In the studied gate, 46.26% of the leech nerve cells are stained as shown on the FL1 scale on the left. The use of the monoclonal antibody did not affect the dot plot profile of the cells. **(C) **On the right, we represent an FSC versus SSC dot plot for leech nerve cells incubated overnight with 8 μl of r*Hm*C1q supernatant, then treated with fluorescein-labeled mouse monoclonal anti-human C1qBP antibody for 30 minutes (dilution 1:250 in L-15 medium). The fluorescence rapidly decreased and only 5.90% of the nerve cells are stained on the FL1 scale on the left. **(D) **A flow cytometry histogram overlay shows a shift in fluorescence when the nerve cells are incubated with anti-human C1qBP antibody (red) compared to either nerve cells that were previously incubated with r*Hm*C1q supernatant (blue) or nerve cells alone (gray). These data are representative of three independent experiments. **(E) **Western blot analysis using polyclonal anti-human C1qBP antibodies from affinity enrichment with biotinylated human C1q. A specific signal corresponding to a 33-kDa molecule, according to the protein ladder (L), was detected when biotinylated human C1q was incubated with leech microglia protein extracts and then eluted on a streptavidin column (1). No immunopositive signal was observed when leech microglial protein extracts were eluted alone on streptavidin column (2) or when biotinylated human C1q was eluted alone with the streptavidin column (3). No signal was detected using the secondary antibody alone as a negative control (data not shown).

Flow cytometry analyses were then carried out in the presence of a monoclonal anti-human C1qBP antibody (Figure 5A-D). A dot plot of leech nerve cells incubated overnight in L-15 medium was deduced (Figure 5A, on the right). Among the selected gate that fits on cells having parameters corresponding to those of microglial cells, 2.86% of the nerve cells (shown in green on the dot plot) were autofluorescent (Figure 5A, on the left). When cells were exposed to the fluorescein-labeled mouse monoclonal anti-human C1qBP antibody, 46.26% were positive (Figure 5B, shown in red on the dot plot and FL1 scale). This antibody recognized a leech protein localized on the cell surface and did not affect the cell dot plot overview. However, when the nerve cells were preincubated overnight with the purified r*Hm*C1q, the number of immunopositive cells strongly decreased to 5.90% (Figure 5C, on the right for the dot plot and left for FL1 scale). Indeed, flow cytometry histogram overlays clearly showed the fluorescence shift observed when the leech cells were preincubated with r*Hm*C1q and exposed to anti-human C1qBP compared to the cell incubation with the anti-human C1qBP alone (Figure 5D). These results show that r*Hm*C1q strongly competed for the target protein specifically recognized by the anti-human C1qBP antibody and suggest that C1qBP might be the *Hm*C1q binding protein.

Because the above results strongly suggested an interaction between *Hm*C1q and a C1qBP, copurification experiments were performed. Preliminary attempts with r*Hm*C1q were undertaken. However, because r*Hm*C1q autoaggregated within some steps of the biotinylation protocol, human C1q was preferred. Indeed, human C1q does not present any massive aggregation and was shown to exhibit a chemotactic effect on leech microglia [17], similarly to r*Hm*C1q. Once biotinylated, human C1q was incubated with leech microglia protein extracts. Following elution on an activated streptavidin column, the interactants of C1q were analyzed by western blotting using polyclonal anti-human C1qBP antibodies (Figure 5E). The polyclonal anti-human C1qBP antibodies specifically recognized a unique 33-kDa molecule (Figure 5E, lane 1), which corresponds to the predicted molecular weight of *Hm*C1qBP. In the first negative control, when microglia protein extracts were incubated on a streptavidin column alone (Figure 5E, lane 2) no signal was obtained, showing that leech microglial proteins cannot recognize streptavidin. In the second negative control, when the sulfo-NHS-SS-biotin-labeled human C1q was loaded alone on streptavidin column (Figure 5E, lane 3), no immunoreactivity was detected, indicating that anti-human C1qBP antibodies do not react with the human C1q. Therefore, these results gave evidence of a specific interaction between human C1q and a C1q-binding protein present in leech microglia protein extract.

## Discussion

In mammals, microglial cells are regulators of tissue homeostasis and are involved in pathological processes orchestrating tissue remodeling. They are currently considered to function as sensors in the brain [28]. Among the mediators expressed by microglial cells and neurons, the subunit C1q belonging to C1 complement factor seems to be a key molecule in neuroinflammatory diseases [29-33]. This complement protein is involved in a large array of vital functions such as the modulation of various immune cells, clearance of apoptotic cells and unwanted synapses, phagocytosis and chemotaxis [34-36]. In several neurodegenerative diseases, tight interactions between C1q and microglial cells may be crucial in the regulation of neuroinflammation [37]. C1q biosynthesis rapidly increases when microglial cells are activated. C1q might be thus considered as a reliable marker for microglia activation.

Of interest in vertebrates, soluble C1q appears to be a potent chemoattractant factor. Indeed, it recruits human immature dendritic cells (DC), neutrophils, eosinophils and mast cells [18,38] through a C1q receptor-dependent mechanism [18,38]. The globular C1q-binding proteins (also called C1qBP, gC1qR, p32, p33 or TAP) interact with the globular heads of C1q and participate in C1q-mediated chemotaxis of human neutrophils [39], human eosinophils [40] and murine mast cells [19]. However, a specific chemotactic activity of C1q through a C1qBP has not previously been reported for mammalian nerve cells.

In the medicinal leech, previous reports have demonstrated that microglial cell recruitment is essential for efficient repair of injured CNS tissue [14]. In this original report we elucidate the role of *Hm*C1q as a chemotactic factor by using a recombinant protein and by identifying a C1q-binding protein (*Hm*C1qBP) as a potential receptor present on microglial cells. This study in the medicinal leech is the first evidence of the interaction between C1q domain-containing protein and C1qBP enabling microglia recruitment in injured CNS.

In the first part of the present study, recombinant *Hm*C1q was produced in the yeast *P. pastoris*, an organism that can be easily manipulated at the molecular genetic level and may express proteins at high levels, intracellularly or extracellularly. In addition, *P. pastoris *performs 'higher eukaryotic' protein modifications, such as glycosylation, disulfide bond formation, and proteolytic processing, compared to bacteria such as *Saccharomyces cerevisiae *or baculovirus [41]. The selected clone studied in this report was immunopositive using anti-*Hm*C1q antibodies, with three distinct immunoreactive bands located in the region corresponding to that of native *Hm*C1q (32.6 kDa). As usually observed in recombinant protein production, there was a slight shift in the final recombinant protein size that can be easily caused by differences in the number and type of added sugar units. Indeed, *P. pastoris *is able to add both *O*-linked and *N*-linked carbohydrate moieties to secreted proteins. Of interest, the recombinant *Hm*C1q exhibited chemotactic activity toward leech microglial cells similar to that of *Hm*C1q-containing medium, previously shown to act as human C1q [17]. A similar dose-dependent chemotactic effect was observed with r*Hm*C1q supernatant as well as with purified r*Hm*C1q (data not shown) and, in these two cases, microglial cell recruitment was specifically neutralized when cells were preincubated with anti-*Hm*C1q antibodies. It must be underlined that (i) microglial recruitment was normal when the cells were preincubated with rabbit preimmune serum, and (ii) control yeast supernatant, without r*Hm*C1q, did not exhibit any chemotactic effect. Therefore, this study shows that the chemotactic effect is dependent on the presence of r*Hm*C1q.

Importantly, *Hm*C1q exhibits an *in vitro *chemotactic effect only on a fraction of microglial cells, suggesting the existence of a subpopulation that is *Hm*C1q-dependent. We have recently shown that crushed nerve cord-conditioned medium contains another chemoattractant factor, homologous to the mature form of interleukin (IL)-16 and named *Hm*IL-16, which also promotes microglial cell migration [22]. As observed for *Hm*C1q, *Hm*IL-16-dependent recruitment is limited to some microglial cells. Therefore, in leech, the involvement of several activation and migration signals acting on different subsets of microglial cells at the lesion site could be taken into account as suggested for mammals [3,42].

Additional *ex vivo *experiments in injured nerve cords have clearly shown that the recombinant *Hm*C1q conserves its functional properties in the whole nerve cord. Indeed preinjection of r*Hm*C1q into injured nerve cords enhances microglial cell migration at the lesion site (r*Hm*C1q vs PBS) and this accumulation is specifically inhibited by anti-*Hm*C1q antibodies but not by preimmune serum. Taken together, these results emphasize the *in vitro *and *ex vivo *chemotactic activity of r*Hm*C1q in the recruitment of resident microglial cells present in leech CNS. In order to specify the *in vitro *chemotactic mechanisms of r*Hm*C1q on microglia, we first used antibodies against a potential C1q receptor as described in the literature [43]. Under our experimental conditions, *Hm*C1q-dependent cell accumulation was abrogated after cell preincubation with the anti-human C1qBP antibody, but not with the isotype control. Therefore it must be assumed that this *Hm*C1q-dependent chemotaxis involves a homolog of human C1q-binding protein (C1qBP, alias gC1qR).

To reinforce this observation, in the second part of the study, attempts were undertaken to characterize the potential receptor of *Hm*C1q on the surface of leech microglial cells. This goal was achieved through several experimental approaches: the identification of a C1qBP-related sequence in leech CNS EST databases, the localization of the leech form of C1qBP, binding competition of *Hm*C1q for the protein specifically recognized by an anti-C1qBP antibody, and the purification of this binding protein to a biotinylated human C1q.

A 33 kDa C1qBP-related molecule was characterized from leech CNS EST databases. This sequence contains a MAM33 domain, which is attributed to an acidic protein of the mitochondrial matrix involved in oxidative phosphorylation and specifically related to the human complement receptor C1qBP [44]. The leech molecule named *Hm*C1qBP exhibits strong similarities with vertebrate and invertebrate known C1qBPs. The comparison between the leech form of C1qBP (*Hm*C1qBP) and the human and mouse ones shows the presence of key residues described as essential for receptor folding and ligand binding properties [27] Most of them (seven out of nine) are perfectly conserved. The residues Thr-137 and Met-239 in the leech sequence are respectively related to residues Lys-123 and Leu-231 in the human one, showing comparable physicochemical features.

Interestingly, the fluorescence *in situ *hybridization led to us specifically locating *Hm*C1qBP mRNA in microglial cells. Following lesions in the leech nerve cord, a timecourse analysis of C1qBP immunostaining was performed using anti-human C1qBP antibodies in order to localize the *Hm*C1qBP protein. The accumulation of microglia was simultaneously observed using Hoechst dye because only microglial cells are able to circulate inside the connectives.

Immediately after a lesion, no specific C1qBP staining was observed in the damaged connectives while no microglial cells accumulated at the crush site. Of interest, over the timecourse analysis (0, 6 and 24 h following the crush) the C1qBP molecule was progressively detected at the lesion site. This increase in cell *Hm*C1qBP staining was exclusively observed where Hoechst-dyed microglia accumulated. Analysis of the lesion site with high magnification revealed, at 6 h following the lesion, that *Hm*C1qBP protein is only present in microglia. Importantly, only a part of the numerous Hoechst-dyed recruited microglial cells are *Hm*C1qBP positive. Therefore, these data suggest that *Hm*C1qBP could be involved in specific recruitment of a well defined microglial cell subpopulation whose chemotaxis is mediated by recognition between *Hm*C1q and *Hm*C1qBP.

Subsequent competition binding assays analyzed using flow cytometry revealed that recombinant *Hm*C1q may share the same protein target recognized by the anti-human C1qBP antibody on the surface of leech microglial cells. Indeed, whereas the anti-human C1qBP antibody alone was found to bind to at least 46% of microglial cells, preincubation with r*Hm*C1q markedly decreased the percentage of cells with specific fluorescence to 5%. This observation shows that r*Hm*C1q acts as a competitor for anti-C1qBP antibodies, indicating that both molecules bind to the same ligand.

To definitively demonstrate that a C1qBP is implicated in leech microglial cell recruitment, a copurification strategy was undertaken using biotinylated human C1q. From the specific complexes eluted, a 33-kDa molecule was specifically detected by western blotting. Interestingly, this product presented the same molecular weight as the predicted *Hm*C1qBP protein. This result, in conjunction with the *in vitro *competitive effect between *Hm*C1q and anti-human C1qBP antibodies, confirms that *Hm*C1q is able to bind to a C1qBP-related molecule in leech microglia. Therefore the present report demonstrates that *Hm*C1qBP has structural and functional analogies with its human counterpart. Though consistent data are provided in this report that clearly demonstrate the involvement of a C1q-binding protein acting as a receptor for *Hm*C1q, the involvement of other potential receptors in microglial cell recruitment during nerve injury cannot be excluded and requires further experiments.

Indeed, in mammals, the C1qBP molecule interacts with the globular heads of C1q [19,45] whereas the other type of C1q-binding protein, the cC1qR (calreticulin) binds to the collagenous portion of C1q [46]. Human C1q has been shown to be a chemotactic factor for human immature dendritic cells. This migration is mediated through ligation of both C1qBP (gC1qR) and cC1qR [18,47]. As the collagen-like sequence is present in *Hm*C1q [17], additional experiments will investigate the existence of a cC1qR-related molecule that might be involved in *Hm*C1q-dependent microglia recruitment.

## Conclusions

Recombinant *Hm*C1q was demonstrated to exert efficient chemoattractant activity *in vitro *and in injured nerve cords. In addition, *Hm*C1q was shown to act on a microglial cell subpopulation through *Hm*C1qBP. In mammals, such an interaction was identified in dendritic cells, but has never been shown in nerve cells. In summary, the production of r*Hm*C1q and the evidence of the involvement of *Hm*C1qBP contribute to a better understanding of microglial activation leading to leech nerve repair. These data from the leech CNS highlight C1q domain-containing factor functions in the integrity of the CNS, as recently suggested in mammals [36,48].

## Competing interests

The authors declare that they have no competing interests.

## Authors' contributions

MT performed experiments for production and purification of recombinant protein, *in vitro *and *ex vivo *chemotaxis assays, flow cytometry and cell binding analyses and participated in manuscript drafting. AGB performed experiments for purification of recombinant protein, cell chemotaxis, cell protein extraction and C1q-binding protein identification and participated in manuscript drafting. FC took part in the cell experiment management, in the interpretation of cell data, in the preparation and in the revision of the manuscript draft. JV took part in cell analysis by immunochemistry and in the images acquisition, and contributed to the manuscript drafting process. PES helped in purification of recombinant protein and western blotting, and contributed to the manuscript drafting process. CV took part in leech cell preparation, in cell chemotaxis, immunohistochemistry and FISH experiments. MS took part in HPLC experiment design and sequence analysis. PNLM was involved in the design of recombinant strategy, in the plasmid construction and all phases of the production of recombinant protein, and participated in manuscript drafting. JP took part in the production of recombinant protein, participated in the conception and coordination of the study design, in the preparation and in the revision of the manuscript draft. CL took part in the sequence analyses, participated in the conception and coordination of the study design, in the preparation and the revision of the manuscript draft. All authors read and approved the final manuscript.

## References

[B1] Olson JK Miller SD Microglia initiate central nervous system innate and adaptive immune responses through multiple TLRs J Immunol 2004 173 3916 3924 15356140 10.4049/jimmunol.173.6.3916

[B2] Gehrmann J Microglia: a sensor to threats in the nervous system? Res Virol 1996 147 79 88 10.1016/0923-2516(96)80220-2 8901425

[B3] Hanisch UK Kettenmann H Microglia: active sensor and versatile effector cells in the normal and pathologic brain Nat Neurosci 2007 10 1387 1394 10.1038/nn1997 17965659

[B4] Farber K Cheung G Mitchell D Wallis R Weihe E Schwaeble W Kettenmann H C1q, the recognition subcomponent of the classical pathway of complement, drives microglial activation J Neurosci Res 2009 87 644 652 10.1002/jnr.21875 18831010 PMC2635544

[B5] Speth C Dierich MP Gasque P Neuroinvasion by pathogens: a key role of the complement system Mol Immunol 2002 38 669 679 10.1016/S0161-5890(01)00104-3 11858822

[B6] Lynch NJ Willis CL Nolan CC Roscher S Fowler MJ Weihe E Ray DE Schwaeble WJ Microglial activation and increased synthesis of complement component C1q precedes blood-brain barrier dysfunction in rats Mol Immunol 2004 40 709 716 10.1016/j.molimm.2003.08.009 14644096

[B7] Mariani MM Kielian T Microglia in infectious diseases of the central nervous system J Neuroimmune Pharmacol 2009 4 448 461 10.1007/s11481-009-9170-6 19728102 PMC2847353

[B8] Duan Y Panoff J Burrell BD Sahley CL Muller KJ Repair and regeneration of functional synaptic connections: cellular and molecular interactions in the leech Cell Mol Neurobiol 2005 25 441 450 10.1007/s10571-005-3152-x 16047551 PMC11529635

[B9] Mladinic M Muller KJ Nicholls JG Central nervous system regeneration: from leech to opossum J Physiol 2009 587 2775 2782 10.1113/jphysiol.2009.169938 19525562 PMC2718237

[B10] Coggeshall RE Fawcett DW The fine structure of the central nervous system of the leech, *Hirudo medicinalis* J Neurophysiol 1964 27 229 289 14129772 10.1152/jn.1964.27.2.229

[B11] Chen A Kumar SM Sahley CL Muller KJ Nitric oxide influences injury-induced microglial migration and accumulation in the leech CNS J Neurosci 2000 20 1036 1043 10648709 10.1523/JNEUROSCI.20-03-01036.2000 PMC6774175

[B12] Kumar SM Porterfield DM Muller KJ Smith PJ Sahley CL Nerve injury induces a rapid efflux of nitric oxide (NO) detected with a novel NO microsensor J Neurosci 2001 21 215 220 11150338 10.1523/JNEUROSCI.21-01-00215.2001 PMC6762443

[B13] von Bernhardi R Muller KJ Repair of the central nervous system: lessons from lesions in leeches J Neurobiol 1995 27 353 366 10.1002/neu.480270308 7673894

[B14] Ngu EM Sahley CL Muller KJ Reduced axon sprouting after treatment that diminishes microglia accumulation at lesions in the leech CNS J Comp Neurol 2007 503 101 109 10.1002/cne.21386 17480028

[B15] Elliott EJ Muller KJ Sprouting and regeneration of sensory axons after destruction of ensheathing glial cells in the leech central nervous system J Neurosci 1983 3 1994 2006 6619920 10.1523/JNEUROSCI.03-10-01994.1983 PMC6564562

[B16] Masuda-Nakagawa LM Muller KJ Nicholls JG Accumulation of laminin and microglial cells at sites of injury and regeneration in the central nervous system of the leech Proc Biol Sci 1990 241 201 206 10.1098/rspb.1990.0086 1979445

[B17] Tahtouh M Croq F Vizioli J Sautiere PE Van Camp C Salzet M Daha MR Pestel J Lefebvre C Evidence for a novel chemotactic C1q domain-containing factor in the leech nerve cord Mol Immunol 2009 46 523 531 10.1016/j.molimm.2008.07.026 18952286

[B18] Vegh Z Kew RR Gruber BL Ghebrehiwet B Chemotaxis of human monocyte-derived dendritic cells to complement component C1q is mediated by the receptors gC1qR and cC1qR Mol Immunol 2006 43 1402 1407 10.1016/j.molimm.2005.07.030 16140380

[B19] Ghebrehiwet B Lim BL Peerschke EI Willis AC Reid KB Isolation, cDNA cloning, and overexpression of a 33-kD cell surface glycoprotein that binds to the globular "heads" of C1q J Exp Med 1994 179 1809 1821 10.1084/jem.179.6.1809 8195709 PMC2191527

[B20] Briand L Perez V Huet JC Danty E Masson C Pernollet JC Optimization of the production of a honeybee odorant-binding protein by *Pichia pastoris* Protein Expr Purif 1999 15 362 369 10.1006/prep.1998.1027 10092496

[B21] Tastet C Lescuyer P Diemer H Luche S van Dorsselaer A Rabilloud T A versatile electrophoresis system for the analysis of high- and low-molecular-weight proteins Electrophoresis 2003 24 1787 1794 10.1002/elps.200305400 12783456 PMC2779374

[B22] Croq F Vizioli J Tuzova M Tahtouh M Sautiere PE Van Camp C Salzet M Cruikshank WW Pestel J Lefebvre C A homologous form of human interleukin 16 is implicated in microglia recruitment following nervous system injury in leech *Hirudo medicinalis* Glia 2010 58 1649 1662 10.1002/glia.21036 20578037

[B23] Kohidai L Method for determination of chemoattraction in *Tetrahymena pyriformis* Curr Microbiol 1995 30 251 253 10.1007/BF00293642 7765899

[B24] Nardelli-Haefliger D Shankland M Lox2, a putative leech segment identity gene, is expressed in the same segmental domain in different stem cell lineages Development 1992 116 697 710 1363227 10.1242/dev.116.3.697

[B25] Altschul SF Madden TL Schaffer AA Zhang J Zhang Z Miller W Lipman DJ Gapped BLAST and PSI-BLAST: a new generation of protein database search programs Nucleic Acids Res 1997 25 3389 3402 10.1093/nar/25.17.3389 9254694 PMC146917

[B26] Marchler-Bauer A Anderson JB Chitsaz F Derbyshire MK DeWeese-Scott C Fong JH Geer LY Geer RC Gonzales NR Gwadz M He S Hurwitz DI Jackson JD Ke Z Lanczycki CJ Liebert CA Liu C Lu F Lu S Marchler GH Mullokandov M Song JS Tasneem A Thanki N Yamashita RA Zhang D Zhang N Bryant SH CDD: a Conserved Domain Database for the functional annotation of proteins Nucleic Acids Res 2011 39 D225 D229 10.1093/nar/gkq1189 21109532 PMC3013737

[B27] Jiang J Zhang Y Krainer AR Xu RM Crystal structure of human p32, a doughnut-shaped acidic mitochondrial matrix protein Proc Natl Acad Sci USA 1999 96 3572 3577 10.1073/pnas.96.7.3572 10097078 PMC22335

[B28] Kreutzberg GW Microglia: a sensor for pathological events in the CNS Trends Neurosci 1996 19 312 318 10.1016/0166-2236(96)10049-7 8843599

[B29] Fonseca MI Chu SH Berci AM Benoit ME Peters DG Kimura Y Tenner AJ Contribution of complement activation pathways to neuropathology differs among mouse models of Alzheimer's disease J Neuroinflammation 2011 8 4 10.1186/1742-2094-8-4 21235806 PMC3033336

[B30] Benoit ME Tenner AJ Complement protein C1q-mediated neuroprotection is correlated with regulation of neuronal gene and microRNA expression J Neurosci 2011 31 3459 3469 10.1523/JNEUROSCI.3932-10.2011 21368058 PMC3080046

[B31] Fraser DA Pisalyaput K Tenner AJ C1q enhances microglial clearance of apoptotic neurons and neuronal blebs, and modulates subsequent inflammatory cytokine production J Neurochem 2010 112 733 743 10.1111/j.1471-4159.2009.06494.x 19919576 PMC2809134

[B32] Flierman R Daha MR Pathogenic role of anti-C1q autoantibodies in the development of lupus nephritis-a hypothesis Mol Immunol 2007 44 133 138 10.1016/j.molimm.2006.06.010 16870257

[B33] Trendelenburg M Antibodies against C1q in patients with systemic lupus erythematosus Springer Semin Immunopathol 2005 27 276 285 10.1007/s00281-005-0007-y 16189648

[B34] Chu Y Jin X Parada I Pesic A Stevens B Barres B Prince DA Enhanced synaptic connectivity and epilepsy in C1q knockout mice Proc Natl Acad Sci USA 2010 107 7975 7980 10.1073/pnas.0913449107 20375278 PMC2867906

[B35] Nauta AJ Castellano G Xu W Woltman AM Borrias MC Daha MR van Kooten C Roos A Opsonization with C1q and mannose-binding lectin targets apoptotic cells to dendritic cells J Immunol 2004 173 3044 3050 15322164 10.4049/jimmunol.173.5.3044

[B36] Nayak A Ferluga J Tsolaki A Kishore U The non-classical functions of the classical complement pathway recognition subcomponent C1q Immunol Lett 2010 131 139 150 10.1016/j.imlet.2010.03.012 20381531

[B37] Tahtouh M Croq F Lefebvre C Pestel J Is complement good, bad, or both? New functions of the complement factors associated with inflammation mechanisms in the central nervous system Eur Cytokine Netw 2009 20 95 100 19825517 10.1684/ecn.2009.0157

[B38] Liu S Wu J Zhang T Qian B Wu P Li L Yu Y Cao X Complement C1q chemoattracts human dendritic cells and enhances migration of mature dendritic cells to CCL19 via activation of AKT and MAPK pathways Mol Immunol 2008 46 242 249 10.1016/j.molimm.2008.08.279 18838169

[B39] Leigh LE Ghebrehiwet B Perera TP Bird IN Strong P Kishore U Reid KB Eggleton P C1q-mediated chemotaxis by human neutrophils: involvement of gClqR and G-protein signalling mechanisms Biochem J 1998 330 247 254 9461517 10.1042/bj3300247 PMC1219134

[B40] Kuna P Iyer M Peerschke EI Kaplan AP Reid KB Ghebrehiwet B Human C1q induces eosinophil migration Clin Immunol Immunopathol 1996 81 48 54 10.1006/clin.1996.0156 8808641

[B41] Cregg JM Cereghino JL Shi J Higgins DR Recombinant protein expression in *Pichia pastoris* Mol Biotechnol 2000 16 23 52 10.1385/MB:16:1:23 11098467

[B42] Prinz M Mildner A Microglia in the CNS: immigrants from another world Glia 2011 59 177 187 10.1002/glia.21104 21125659

[B43] Lim BL Reid KB Ghebrehiwet B Peerschke EI Leigh LA Preissner KT The binding protein for globular heads of complement C1q, gC1qR. Functional expression and characterization as a novel vitronectin binding factor J Biol Chem 1996 271 26739 26744 10.1074/jbc.271.43.26739 8900153

[B44] Seytter T Lottspeich F Neupert W Schwarz E Mam33p, an oligomeric, acidic protein in the mitochondrial matrix of *Saccharomyces cerevisiae *is related to the human complement receptor gC1q-R Yeast 1998 14 303 310 10.1002/(SICI)1097-0061(19980315)14:4<303::AID-YEA217>3.0.CO;2-N 9559539

[B45] Eggleton P Ghebrehiwet B Sastry KN Coburn JP Zaner KS Reid KB Reid KB Tauber AI Identification of a gC1q-binding protein (gC1q-R) on the surface of human neutrophils. Subcellular localization and binding properties in comparison with the cC1q-R J Clin Invest 1995 95 1569 1578 10.1172/JCI117830 7706463 PMC295648

[B46] Erdei A Reid KB Characterization of C1q-binding material released from the membranes of Raji and U937 cells by limited proteolysis with trypsin Biochem J 1988 255 493 499 3144267 PMC1135255

[B47] Hosszu KK Santiago-Schwarz F Peerschke EI Ghebrehiwet B Evidence that a C1q/C1qR system regulates monocyte-derived dendritic cell differentiation at the interface of innate and acquired immunity Innate Immun 2010 16 115 127 10.1177/1753425909339815 19710097 PMC2846191

[B48] Shimono C Manabe R Yamada T Fukuda S Kawai J Furutani Y Tsutsui K Ikenaka K Hayashizaki Y Sekiguchi K Identification and characterization of nCLP2, a novel C1q family protein expressed in the central nervous system J Biochem 2010 147 565 579 10.1093/jb/mvp203 19996152

